# An umbrella review on the use of antipsychotics in anxiety disorders: A registered report protocol

**DOI:** 10.1371/journal.pone.0269772

**Published:** 2022-06-16

**Authors:** Amir Garakani, Rafael C. Freire, Frank D. Buono, Robyn P. Thom, Kaitlyn Larkin, Melissa C. Funaro, Mona Salehi, Mercedes M. Perez-Rodriguez

**Affiliations:** 1 Department of Psychiatry and Behavioral Health, Greenwich Hospital, Greenwich, CT, United States of America; 2 Department of Psychiatry, Yale School of Medicine, New Haven, Connecticut, United States of America; 3 Department of Psychiatry and Centre for Neuroscience Studies, Queen’s University, Kingston, Ontario, Canada; 4 Laboratory of Panic and Respiration, Institute of Psychiatry, Federal University of Rio de Janeiro, Rio de Janeiro, Brazil; 5 Department of Psychiatry, Massachusetts General Hospital, Harvard Medical School, Boston, MA, United States of America; 6 Department of Psychology, Northern Illinois University, DeKalb, IL, United States of America; 7 Harvey Cushing/John Hay Whitney Medical Library, Yale University, New Haven, CT, United States of America; 8 Department of Psychiatry and Behavioral Sciences, Johns Hopkins University School of Medicine, Baltimore, MD, United States of America; 9 Department of Psychiatry, Icahn School of Medicine at Mount Sinai, New York, NY, United States of America; University of Sydney, AUSTRALIA

## Abstract

Anxiety disorders, including panic disorder (PD), generalized anxiety disorder (GAD), social anxiety disorder (SAD), agoraphobia, and specific phobia, are among the most common psychiatric disorders. Although the traditional pharmacologic treatments for anxiety included barbiturates and then benzodiazepines, the introduction of tricyclic antidepressants, followed by the selective serotonin reuptake inhibitors (SSRIs), marked a tidal shift in the treatment of anxiety. Although not approved for treatment of anxiety disorders (with the exception of trifluoperazine) there is ongoing off-label, unapproved use of both first-generation “typical” antipsychotics (FGAs) and second-generation or “atypical” antipsychotics (SGAs) for anxiety. Although there have been systematic reviews and meta-analyses on the use of antipsychotics in anxiety disorders, most of these reviews focused on SGAs, primarily the use of quetiapine in GAD. Given that there is little known about the potential benefits and short-and long-term risks of using antipsychotics in anxiety, there is a need for an umbrella review of systematic reviews and meta-analyses of the use of both FGAs and SGAs in anxiety disorders. The specific aims of this study are as follows: (1) Evaluate the evidence of efficacy of FGAs and SGAs in anxiety disorders as an adjunctive treatment to SSRIs, serotonin norepinephrine reuptake inhibitors (SNRIs) and other non-antipsychotic medications; (2) Compare monotherapy with antipsychotics to first-line treatments for anxiety disorders in terms of effectiveness, risks, and side effects; and (3) Evaluate the short- and long-term risks and side effects of prescribing antipsychotics in anxiety disorders. The review is registered on PROSPERO (CRD42021237436). Since data extraction has not begun, there is not preliminary data to share.

## Introduction

Anxiety disorders represent the most common psychiatric disorders by prevalence in the United States [1]. In the DSM-5, the primary anxiety disorders affecting adults include panic disorder (PD), generalized anxiety disorder (GAD), social anxiety disorder (SAD), agoraphobia, and specific phobia. Although the traditional pharmacologic treatments for anxiety included barbiturates and then benzodiazepines, the introduction of tricyclic antidepressants, followed by the selective serotonin reuptake inhibitors (SSRIs), marked a tidal shift in the treatment of anxiety. Treatment guidelines recommend, as first-line pharmacotherapy, the use of SSRIs or venlafaxine for PD, GAD and SAD [2–4]. Although the use of benzodiazepines continue to be an option, there have been concerns raised about their long-term use, tolerance, dependence, and abuse potential, along with risks of falls; additionally, while there was initial evidence of dementia with chronic benzodiazepine use, a recent nationwide cohort and nesting case-control study of 235,465 patients reported no association between benzodiazepines and subsequent dementia, and allowed for speculation that they may even have protective effects [5]. As a result of negative perceptions, benzodiazepine prescribing has fallen out of favor, despite their clear efficacy especially early in treatment and as an as-needed option for anxiety [6].

Given the efficacy of psychotherapies such as cognitive behavioral therapy (CBT) and exposure therapy, as well as medications such as SSRIs, serotonin norepinephrine reuptake inhibitors (SNRIs), tricyclic antidepressants, pregabalin, and benzodiazepines, there would be ample therapeutic options for anxiety disorders. Despite the presence of treatment options, there has been limited research done on the management of patients with treatment-resistant or refractory anxiety [7]. Such patients are often considered for SNRIs, monoamine oxidase inhibitors (MAOs), other antidepressants, or combination treatments with other anxiolytic agents like the azapirones (e.g., buspirone). In spite of these therapeutic options, between 15–40% of patients with anxiety disorders do not respond (50% improvement to these first-line treatments) and roughly half of patients do not recover (defined as having minimal anxiety symptoms) [7]. There are also high rates of comorbidity between anxiety disorders and mood disorders [8], particularly major depressive disorder (MDD), and evidence that these patients are poorer responders to antidepressant treatment and have greater symptom severity including higher rates of suicide [9]. Antipsychotics represent a class of medications that have come under increased study for non-psychotic disorders over the years. The first-generation “typical” antipsychotics (FGAs) were prescribed primarily for schizophrenia but also were used for bipolar mania, agitation (psychotic and non-psychotic), sleep induction and Tourette’s syndrome. One antipsychotic, trifluoperazine, was also approved by the US Food and Drug Administration (FDA) in 2001 for non-psychotic anxiety [10], supported by a large randomized-double-blind, placebo-controlled study in 1986 [11]. As of 2020, there are no second-generation or “atypical” antipsychotics (SGAs) approved for any of the anxiety disorders. There is, however, ongoing use of both FGAs and SGAs for disorders other than psychotic and bipolar disorders, which is considered “off-label” (i.e., not approved by FDA or equivalent agency) [12,13]. Data collected on inpatients and outpatients in an academic setting suggest that prescriptions of antipsychotics for anxiety and anxiety disorders is common [14]. Previous systematic reviews report that there is evidence, in the form of randomized, double-blind, placebo-controlled trials, for the use of some SGAs in certain anxiety disorders [15,16]. Canadian guidelines [4] recommend antipsychotics only as a third-line option or adjunctive therapy for anxiety disorders, which may include olanzapine, quetiapine, risperidone, or aripiprazole, depending on the anxiety disorder. There is limited to poor support for the use of SGAs in PD [17]. These reviews suggest that the evidence is strongest for the use of quetiapine in GAD, but caution is advised given the poor tolerability and potential for serious adverse effects [18–20].

To date, despite the absence of a formal approval, antipsychotics continue to be prescribed off-label for various indications including insomnia and anxiety. There may be increasing use of antipsychotics in anxiety in cases where patients have been refractory to first- or second-line treatment options. The Canadian Network for Mood and Anxiety Treatments (CANMAT) Guidelines recommend SGAs both as treatment for bipolar disorder and (mostly as adjunctive for) MDD [21,22]. Patients with anxiety disorders and comorbidity with bipolar disorder or MDD are likely to receive SGAs as part of their treatment because of these recommendations. In addition, inferences that medications considered effective for MDD are also effective for anxiety disorders can increase the prescription of SGAs for patients with anxiety. The concern arises that there may not be sufficient evidence to support the use of this class of medications and there may not be sufficient consideration given to the short- and long-term risks of use of these medications, especially in vulnerable populations (e.g., children, adolescents, and those who are medically ill or immunocompromised, elderly and cognitively impaired). Clinically, medical providers prescribing antipsychotics for anxiety may choose to do so in cases where patients have not responded to first- and second-line treatments or have refractory symptoms, as long as they provide psychoeducation about the risks and benefits and potential adverse reactions such as tardive dyskinesia, neuroleptic malignant syndrome, metabolic syndrome (weight gain, elevated lipids, development of diabetes), extrapyramidal symptoms, and cardiac effects including arrhythmias and sudden death. Patients given such information may choose to forego treatment with SGAs or FGAs. That said, there is a widespread use of antipsychotics in primary care, less than a half of the prescriptions are for bipolar disorder or psychotic disorders [13]. A significant portion of antipsychotic prescriptions in primary care is for anxiety disorders [13], SGAs and FGAs are also prescribed for anxiety in other clinical settings. The concern arises about the risk of prescribing such medications to patients long-term and in particular higher-risk populations like children, those medically ill and the elderly. A recent study reported greater mortality in patients with dementia receiving both antipsychotics and benzodiazepines [23] and in patients with schizophrenia receiving this drug combination [24]. There is even evidence for increased mortality risk in otherwise-healthy adult patients with depression receiving antipsychotic augmentation of their antidepressant [25]. Additionally, the rates of tardive dyskinesia, while lower with SGAs (21%) compared to FGAs (30%), still remain high for such a serious and irreversible drug reaction [26].

There is a strong need for a review of the literature on the use of both FGAs and SGAs in anxiety disorders, as most of the current systematic reviews and meta-analyses exclude first-generation antipsychotics [15–20], with a focus on the most common DSM-5 anxiety disorders affecting adults: PD, agoraphobia, specific phobias, GAD and SAD.

### Objectives

The objective of the current study is to conduct an umbrella review of systematic reviews and meta-analyses to evaluate the efficacy and safety of first and second-generation antipsychotics for the treatment of panic disorder, generalized anxiety disorder, specific phobia, social anxiety disorder (social phobia) and agoraphobia.

#### Specific aims

Specific aim 1: Is there evidence of efficacy of first- and second-generation antipsychotics in anxiety disorders as an adjunctive treatment to SSRIs, SNRIs and other non-antipsychotic medications?

Specific aim 2: How does monotherapy with antipsychotics compare to first-line treatments for anxiety disorders (effectiveness, risks, and side effects)?

Specific aim 3: What are the short- and long-term risks and side effects of prescribing antipsychotics in anxiety disorders?

## Methods

The umbrella review protocol is informed by the framework described by the Joanna Briggs Institute [27]. In addition, the Preferred Reporting Items for Systematic Reviews and Meta-Analysis (PRISMA) guidelines will be used throughout the process [28]. A Preferred Reporting Items for Systematic Review and Meta-Analysis Protocols (PRISMA-P) checklist has been completed [29] (see S1 Checklist). This umbrella review protocol has been registered with the International Prospective Register of Systematic Reviews (PROSPERO) (registration number CRD42021237436).

### Inclusion and exclusion criteria

Reviews will be considered even if they are part of a review of the use of antipsychotics in non-anxiety disorders or if the reviews are part of a larger study of pharmacological treatments of anxiety disorders. Reviews of antipsychotic monotherapy in anxiety disorders will be included. Reviews including adjunctive treatments of an antipsychotic with other medications (such as SSRIs, SNRIs, benzodiazepines, etc.) in anxiety disorders will be included as this reflects “real-life” clinical practice. Systematic reviews of adults over the age of 18 years will be included. Reviews in peer-reviewed journals in the English language will be included.

Reviews of use of antipsychotics in anxiety disorders comorbid with other disorders (such as depressive disorders, bipolar disorder, schizophrenia, substance use disorders, medical illnesses, etc.) will be excluded if the anxiety disorder is a secondary diagnosis. Reviews will be included if they comprise patients with comorbidities, but the anxiety disorder is the primary diagnosis. Reviews in children and adolescents under the age of 18 years will be excluded. Non-systematic reviews or narrative reviews or opinion-editorial pieces will be excluded. Reviews in non-peer-reviewed journals that are not in the English language will be excluded.

### Information sources

A systematic search of the following databases will be completed: PubMed, MEDLINE, EMBASE, APA PsycInfo, CINAHL Complete; and the Cochrane Library from inception onwards. The search will be limited by study design using a search filter to identify systematic reviews and meta-analyses in MEDLINE, Embase, CINAHL, and APA PsycInfo following the SIGN recommended search terms for systematic reviews and meta-analyses [30]. Searches will be limited to the English language. No date limits will be imposed on the search.

An experienced medical librarian (MCF) will be consulted on methodology. A medical subject heading (MeSH) analysis of known key articles provided by the research team [mesh.med.yale.edu] will be done and scoping searches performed in each database. An iterative process will be used to translate and refine the searches. To maximize sensitivity, the formal search will use controlled vocabulary terms and synonymous free-text words to capture the concepts of “anxiety disorders” and “antipsychotic agents.” The search strategy will be peer reviewed by a second librarian, not otherwise associated with the project, using the PRESS standard. A draft MEDLINE search strategy is included in Table 1. Reviewers will check for additional relevant cited and citing articles using included studies. To capture recently published articles, a second database search will be rerun before publishing the paper.

**Table 1:** Search strategy ovid MEDLINE® all.

line number	Search
1	Meta-Analysis as Topic/
2	meta analy$.tw.
3	metaanaly$.tw.
4	Meta-Analysis/
5	(systematic adj (review$1 or overview$1)).tw.
6	exp Review Literature as Topic/
7	or/1-6
8	cochrane.ab.
9	embase.ab.
10	(psychlit or psyclit).ab.
11	(psychinfo or psycinfo).ab.
12	(cinahl or cinhal).ab.
13	web of science.ab.
14	or/8-13
15	reference list$.ab.
16	bibliograph$.ab.
17	hand-search$.ab.
18	relevant journals.ab.
19	manual search$.ab.
20	or/15-19
21	selection criteria.ab.
22	data extraction.ab.
23	21 or 22
24	Review/
25	23 and 24
26	Comment/
27	Letter/
28	Editorial/
29	animal/
30	human/
31	29 not (29 and 30)
32	or/26-28,31
33	7 or 14 or 20 or 25
34	33 not 32
35	Anxiety Disorders/ or exp agoraphobia/ or exp neurotic disorders/ or exp panic disorder/ or exp phobic disorders/ or (anxieties or anxiety or acrophobia* or agoraphobia* or claustrophobia* or neuroses or neurosis or neurotic or ophidiophobia* or panic or phobic or phobia or phobias).tw,kf.
36	exp antipsychotic agents/ or risperidone/ or olanzapine/ or olanzapine-fluoxetine combination/ or 9-hydroxy-risperidone/ or dopamine antagonists/ or molindone/ or (antipsychotic* or anti-psychotic* or neuroleptic* or aceperone or acepromazine or aceprometazine or acetophenazine or adoprazine or alimemazine or amisulprid* or (amitriptyline and perphenazine) or amperozide or aplindore or aripiprazole or asenapine or azaperone or balipodect or benperidol or bitopertin or blonanserin or brexpiprazole or bromospiperone or bromperidol or butaclamol or butaperazine or carfenazine or cariprazine or carpipramine or centbutindole or chlorphenethazine or chlorproethazine or chlorpromazine or chlorprothixene or cinuperone or clocapramine or clofluperol or clopenthixol or clopimozide or clospipramine or clothiapine or clotiapine or clozapine or cyamemazine or dapiprazole or deutetrabenazine or dicarbine or dimetotiazine or dixyrazine or droperidol or ecopipam or etazolate or fananserin or farampator or fencamfamine or fluanisone or flupenthixol or flupentixol or fluperlapine or fluphenazine or fluspirilene or flutroline or gevetroline or haloperidol or hioproperazine or iloperidone or isofloxythepin or isomolpan or landipirdine or lenperone or levomepromazine or loxapine or lumateperone or lurasidone or mardepodect or maroxepine or mazapertine or melperone or mepiprazole or mesoridazine or methiothepin or methopromazine or methotrimeprazine or metofenazate or metylperon or molindone or moperone or mosapramine or nemonapride or nialamide or noctran or norchlorpromazine orolanzapine or (olanzapine and fluoxetine) or operone or oxiperomide or oxypertine or oxyprothepine or paliperidone or pecazine or penfluridol or perazine or pericyazine or perimetazine or perospirone or perphenazine or picobenzide or piflutixol or pimavanserin or pimethixene or pimozide or pipamperone or piperacetazine or pipotiazine or pirenperone or pomaglumetad or prochlorperazine or profenamine or promazine or propericiazine or propiomazine or propionylpromazine or prothipendyl or quetiapine or raclopride or remoxipride or reserpine or rimcazole or risperidone or ritanserin or roluperidone or savoxepine or sertindole or setoperone or spiperone or spiroperidol or stepholidine or sulforidazine or sulpiride or sultopride or tefludazine or tetrabenazine or tetrahydropalmatine or thiopropazate or thioproperazine or thioridazine or thiothixene or tiapride or timiperone or tiotixene or (tranylcypromine and trifluoperazine) or triethylperazine or trifluoperazine or trifluperidol or triflupromazine or umespirone or vabicaserin or veralipride or zetidoline or zicronapine or ziprasidone or zoloperone or zotepine or zuclopenthixol).tw,kw.
37	34 and 35 and 36
38	limit 37 to English language

### Study selection

Search results will be pooled in EndNote 20 and de-duplicated [www.endnote.com]. This set will be uploaded to Covidence [www.covidence.org] for screening.

Two screeners will independently review the titles, abstracts and full-text of the eligible articles using Covidence. The studies will be graded on the measure of either having criteria “met” or “not met” with the latter studies being excluded. For cases where there is a lack of certainty, there will be an option for “not clear.” These studies will then be assessed by the third reviewer (AG) and a consensus will be reached after discussion between the reviewers after the initial review. Studies will be selected or excluded after a full-text review by two board-certified psychiatrists (AG and RCF) who are blinded to each other and working independently. Any discrepancy will be resolved by discussion between the two reviewers, or with a third person (RPT) if consensus cannot be reached.

### Data collection

Data on study characteristics will be extracted independently, by two reviewers, using a standardized, pre-piloted JBI data extraction form. Disagreements will be resolved through discussion with a third reviewer to reach consensus. Reviewers will extract quantitative and qualitative data from each selected study, grouped by the type of intervention. Findings will be presented in tabular format with supporting text. Quantitative tabulation of results will include: first author name and year of publication; setting; characteristics of the study population; number of RCTs included in the systematic review; findings related to the intervention/s and comparator regimens used; the outcomes assessed.

To reduce bias, the methodological quality of the systematic reviews and meta-analysis will be evaluated using the AMSTAR-2 (A MeaSurement Tool to Assess systematic Reviews) scale [31], and a global score will be computed awarding a point for each domain that can be rated as yes/partially yes. We will also extract data about the funding of each systematic review/meta-analysis and the conflicts of interest of the authors, focused on any potential financial benefits associated to the intervention.

### Data analysis

Each systematic review and meta-analysis will be re-analyzed with a random effects model, including estimations of heterogeneity (τ^2^ and I^2^) and prediction intervals. The unit of analysis will be conducted through qualitative review across the reviewers, a standard and validated approach [32]. Small study effects will be examined with the Egger test for each systematic review and meta-analysis with over 10 studies [33]. We will compute estimations of the aggregated effect which can be regarded as free from small sample bias by extrapolating the Egger regression line on a funnel plot to a theoretical study of infinite sample size and a standard error of 0 [34]. To ensure no overlapping reviews are in this analysis, we will implement a corrected covered analysis [35]. A CCA will be measure the overlap by the frequency of repeated occurrences of the indexed publication, to evaluate possible range of CCA for each review. We will also apply the excess significance test, which quantifies whether the observed number of studies with statistically significant results (“positive studies") differs from the expected number of positive studies, considering the estimates of statistical power for each study included in the systematic review and meta-analysis [36–38]. If articles are unpublished at the point of extraction, we will implement a Bayesian approach to account for uncertainty in the analysis. Using a procedure adapted from existent umbrella reviews [32], we will identify outcomes and interventions for which summary effects showed strong evidence of significance (p<0.001); were based on more than 1000 randomized participants; did not have large heterogeneity between studies (I^2^ <75%); their 95% prediction interval excluded the null; extrapolation of the Egger regression line for infinite sample size still suggested a nominally significant effect (i.e., 95% confidence interval did not include the null); had no evidence for excess significance; came from systematic reviews and meta-analysis with a score of at least 9 on the AMSTAR-2 scale; came from systematic reviews and meta-analysis not funded by the entity developing or marketing the intervention and free from authors with a financial conflict of interest associated to the intervention.

## Supporting information

S1 Checklist (DOCX)
